# Trends in usage and regional variations in Ethiopian animal feeding practices: A 15-year analysis for informed policy

**DOI:** 10.1371/journal.pone.0338019

**Published:** 2026-02-09

**Authors:** Desalegn Begna, Zemene Yohannes, Netsanet Jote, Girma Teshome, Alemnew Mekonen

**Affiliations:** 1 Agricultural and Rural Development Centre, Policy Studies Institute, Addis Ababa, Ethiopia; 2 Industrial Development Policy Studies Centre, Policy Studies Institute, Addis Ababa, Ethiopia; 3 Environmental change and climate pollution, Policy Studies Institute, Addis Ababa, Ethiopia; Jimma University College of Agriculture and Veterinary Medicine, ETHIOPIA

## Abstract

Livestock feeding practices play a pivotal role in sustaining agricultural productivity and food security in Ethiopia. However, the sector continues to face structural challenges, including a heavy reliance on traditional feed resources and pronounced regional disparities. This study utilized secondary data from the Agricultural Sample Surveys conducted annually by the Central Statistical Agency (2004/05–2018/19) to assess feeding practices, examine regional variations, and analyze long-term trends. Descriptive statistics, trend analysis, and correspondence analysis were applied. Different data analytical techniques were employed, including descriptive statistics, trend analysis, and Correspondence Analysis (CA), were employed to monitor livestock feeding practices, examine the regional differences, and explore trends over the long horizon. Results showed a decline in reliance on green fodder (from 40.3% to 36.9%) and crop residues (from 33.5% to 32.0%), accompanied by a modest increase in the use of improved feeds (0.15% to 0.90%) and agro-industrial by-products (2.7% to 4.6%). Regional differences were substantial: Afar predominantly used green fodder (82%), while Harari relied more on crop residues (41%). In high-livestock-population regions such as Amhara, Oromia, and SNNP, feeding practices varied according to resource availability and management strategies. These findings underscore the need for targeted, region-specific interventions to enhance feed availability, promote adoption of sustainable and cost-effective feeding systems, and address persistent demand–supply imbalances. The evidence also offers insights for policy reforms in other agriculture-based developing economies.

## Introduction

In sub-Saharan Africa, including Ethiopia, livestock feed is predominantly sourced from locally available materials, primarily natural products such as grains, forages, and agro-processing by-products [1,2]. The feed sector faces mounting pressure from growing demand for livestock products, driven by rapid population growth, alongside constraints from climate change and limited resources. Ethiopia, the second most populous country in Africa, is projected to grow from over 120 million people today to approximately 200 million by 2050. Rising demand for animal protein, fuelled by urbanization and income growth, presents both opportunities and constraints for agricultural and livestock development. Additionally, demand for animal protein is rising increasing due to accompanying urbanization and income growth which provides both opportunities and constraint for agricultural and livestock development. Maintaining livestock productivity will require sustainable production systems, which fundamentally depend on addressing Ethiopia’s feed challenges to meet the needs of its growing population [3].

Ethiopia possesses a large and genetically diverse livestock population that plays a pivotal role in the nation’s socio-economic development. The government considers the livestock sector a driver of economic growth and promotes investments that stimulate agro-industrial development. The sector generates export revenue from meat, dairy, and leather products, provides employment for millions of rural smallholder farmers, and attracts industrial investment in feed processing plants, slaughterhouses, and value-added processing. By leveraging its rich livestock genetic resources, Ethiopia aims to make the sector a catalyst for economic transformation and sustainable development [4]. However, a persistent feed supply shortage continues to constrain the sector’s performance and limit its contribution to the national economy [5]. Feed is central to livestock growth and transformation, as its availability and quality directly determine production levels, product quality, and economic viability [6,7]. As a limited resource, feed is competed for by all livestock species, underscoring its critical role in achieving economic, social, and environmental objectives [8,9]. Consequently, Ethiopia’s livestock sector exhibits clear signs of feed scarcity, including low milk yields (1.4 liters/day), slow growth rates, prolonged calving and lambing intervals, and delayed maturity manifestations of inadequate feed availability [5]. Traditionally, livestock feeding in Ethiopia has relied on natural forages, agricultural by-products, grazing pastures, stubble feeding (aftermath), and similar resources [8,10–12]. As demand for livestock products continues to grow [13–15], several reports indicate that the sustainability of these traditional feed resources is under threat due to land degradation, farmland expansion, climate variability, and inefficient farming practices [16,17]. However, comprehensive information on long-term (15-year) trends in Ethiopia’s feed landscape is lacking, particularly regarding production dynamics, sourcing patterns, regional variations, and the factors influencing these practices. Previous research has described the livestock sector but offers limited empirical analysis of feed sources and related regional dynamics over this period. This study addresses this gap by measuring changes in feed sources and usage trends from 2004/05–2018/19, and by examining spatial and temporal patterns across Ethiopia’s regions. Specifically, it tracks declines in traditional feed sources, the slow uptake of improved feeds, and significant geographical disparities in feeding practices. Correspondence and trend analyses are used to describe the structural challenges facing Ethiopia’s livestock sector. In sum, the study aims to inform policymakers about Ethiopia’s feed-related needs to meet growing demand for livestock products by increasing feed availability, improving livestock productivity, and promoting sustainable agricultural development.

## Methodology

This study examined Ethiopia’s animal feed growth trajectories, evolving practices, and regional variations over a 15-year period (2004/05–2018/19). These years were chosen based on the availability of consistent data from the Central Statistical Agency of Ethiopia’s annual Agricultural Sample Surveys. The 2004/05 season marked major policy shifts in Ethiopia’s agricultural sector, including increased government emphasis on livestock development. The 2018/19 season was the most recent year with consistent, reliable data, providing a solid temporal window to assess long-term trends and structural shifts in feeding practices. The selection of these cut-off points offer a sound temporal perspective for investigating the array of long-term trends, and the structural shifts in feeding practices in Ethiopia’s livestock sector. The study relied on 15 years of official secondary data from the Ethiopian Statistical Service (formerly Central Statistical Agency), primarily from its Agricultural Sample Surveys. The official nature of these datasets ensures their reliability and relevance to Ethiopia’s livestock sector.

Given the data is secondary, its official nature provides assurance that it is reliable and pertinent to the specific context of Ethiopia´s livestock sector. [18], now the Ethiopian Statistical Service (ESS, 2004–2019). Supplementary data were sourced from peer-reviewed literature, government publications, and reputable NGOs. Data cleaning involved validating completeness, imputing missing values where possible, and discarding unresolvable extreme values. Duplicate entries and statistical outliers were also removed.. A structured cleaning process was used to validate the accuracy and completeness of the dataset. The cleaning process incorporated the consideration of missing values as far as possible with an imputation process, while extreme values were considered unresolvable and their records were discarded. Duplicate entries were also identified through the process of cleaning as well as outlier exclusion.

Feed materials were classified into six categories: green forage (including grazing), crop residues (straw, stalks), improved feed (commercially formulated products), hay (dried grasses or legumes), industrial by-products (e.g., oilseed cakes, molasses, seed-processing residues), and other (e.g., kitchen waste). [19]. Each category was defined using clear operational criteria to ensure consistency and mutual exclusivity.

Statistical techniques included descriptive statistics, trend analysis, and correspondence analysis to examine spatial and temporal variations. R and SPSS were used for statistical analysis, while ArcGIS and QGIS facilitated spatial mapping of regional trends [18].

## Results and discussions

### Overall contributions of all feeding types

**Fig 1:**
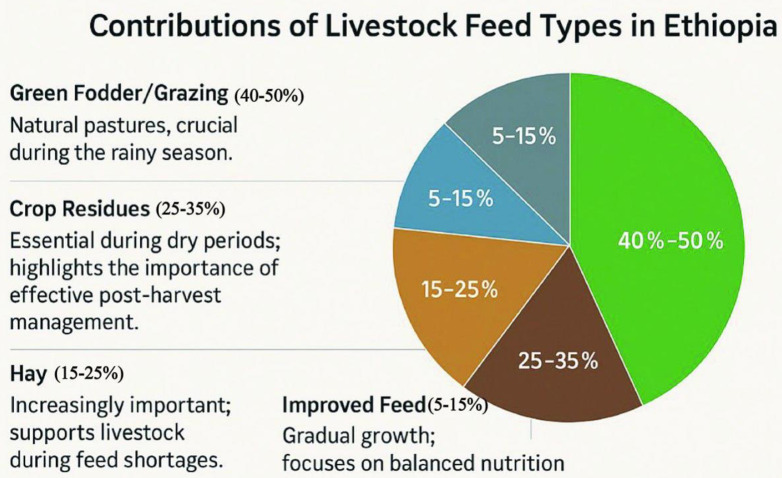
Proportional contributions of livestock feed types in Ethiopia.

The contributions of various livestock feed types in Ethiopia, highlighting their significance in livestock management, are summarized in Fig 1. The figure illustrates the current landscape of feeding practices, identifying dominant and emerging feed sources and underscoring the importance of feed diversity for sustainable livestock production. Trends indicate minimal adoption of improved feeds, suggesting a gap between awareness and practical implementation.

Green fodder/grazing, comprising 40% to 50% of total feed, is the primary feed source. This highlights the vital role of natural pastures, especially during the rainy season when fresh forage is plentiful. The heavy reliance on green fodder emphasizes its key role in sustaining livestock nutrition and productivity [20].

Crop residues contribute approximately 25% to 35% of total feed, especially during dry seasons. Their consistent role is crucial for maintaining livestock nutrition when other feeds are scarce [10,21].

The hay is represented as an increasingly significant feed source, contributing 15% to 25%. This rising trend indicates a shift toward better feed storage practices, allowing farmers to provide essential nutrition for livestock during feed shortages. The recognition of hay’s importance demonstrates an evolving approach to livestock feeding strategies [22].

Although improved feeds constitute a smaller proportion (5% to 15%), their gradual increase indicates growing adoption of nutritionally balanced options aimed at improving livestock health and productivity [23].

The by-products and other feed sources contribute around 5% to 10% of the total supply. Their variable contribution highlights their role as complementary resources to traditional feeds, providing additional nutrients during critical periods [24].

### The feeds-growth trends over the years

**Fig 2:**
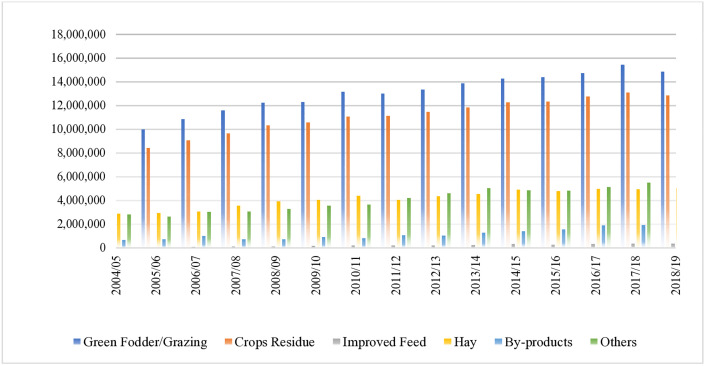
Animal feed-growth by feed sources.

Fig 2 illustrates the growth trends of various feed types over years from 2004/05–2018/19, highlighting the contributions of green fodder/grazing, crop residues, hay, improved feed, by-products, and other feed sources.

Green fodder and grazing have remained the predominant animal feed sources; however, their reported usage declined from 40.29% in 2004/05 to 36.93% in 2018/19. This decrease likely reflects reduced access to natural grazing areas, attributable to land degradation, agricultural expansion, and urbanization, all of which negatively impact livestock nutrition. [25].

#### Green fodder of grazing land.

Results from this 15-year analysis indicate that grazed pasture has always been the leading animal feed source in Ethiopia and it accounted for a considerable share of livestock feed during this reporting period [26]. The data indicate that the amount of feed received from grazing lands has remained fairly constant with an estimated average of over 56% of the total amount of feed supply for mixed livestock systems being contributed by green fodder [26]. The data also emphasizes the lingering dependence on natural grazing places, particularly in rural and pastoral communities where other sources of animal feed may be limited [27].

**Fig 3:**
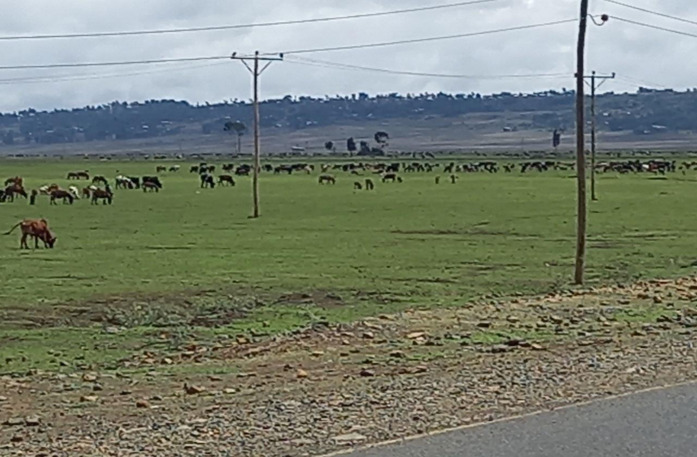
Large number of livestock at freely grazing green fodder.

Although there have been some efforts to promote practices, such as cultivating forages and incorporating crop residues, these interventions have seen minimal uptake [28]. The continuing dominance of green grazing and natural pasture likely stems from several factors [27]. This dependence suggests barriers to adoption, including limited access to improved feeds, extension services and economic barriers that would enable livestock keepers to diversify feeding strategies, potentially leading, amongst other things to increased concentrations of nutrients in feeding systems [28].While the existing literature has value, the findings of the study confirmed that green grazing has always been the primary source of animal feed for a period of 15 years [26]. This suggests that little change has occurred regarding alternative or improved feed sources [28]. It highlights that there have been no significant improvements in feeding systems in the last 15 years that was not made with good intention but little to show for it, highlighting the need for specific interventions to diversify feed sources and management of grazing land. Many strategies, like incorporating improved forages, increasing the productivity of grazing land, and to strengthen extension services could collectively decrease the degree of reliance on green grazing gradually and improve livestock’s productivity and sustainability [29]. Fig 1 shows trends in feed growth, while Fig 3 depicts mixed-species livestock freely grazing on communal lands” confirming that communal grazing lands are essential for feed supply and meeting animals’ nutritional requirements [30,31].

#### Crop residues.

**Fig 4:**
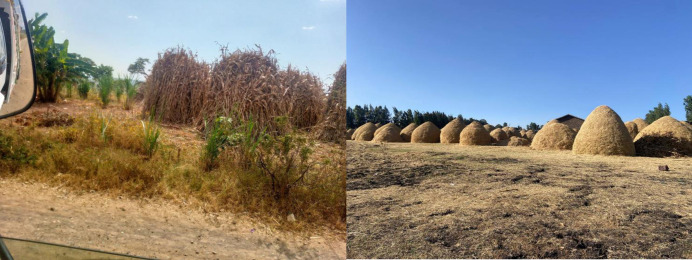
Sorghum and Teff straw piled as crop residue for animal feed.

Crop residues, such as straw, Stover, and husks, are the by-products left after harvesting main grains. They are primarily used as animal feed, especially during dry seasons when fresh forage is scarce [32,33], and their use helps reduce feed costs [34]. In Ethiopia, crop residues (Fig 4) are a critical source of livestock feed, providing essential nutrients when other feed options are limited [35] and management and utilization of residues can significantly impact livestock productivity and health [36]. The number of holders using crop residues as animal feed increased over 15 years, from 8.1 million to 12.8 million. However, between 2017/18 and 2018/19, this number declined slightly, from 13.1 million to 12.8 million holders (Table 1).

**Table 1:** Percentage of livestock holders using various animal feed types in Ethiopia: 15-year trends (2004/05–2018/19).

Years	Total	Green Fodder/Grazing		Crops Residue		Improved Feed		Hay		By-products		Others	
	Number	Number	%	Number	%	Number	%	Number	%	Number	%	Number	%
2004/05	24,300,256	9,789,364	40.29	8,128,882	33.45	37,297	0.15	2,874,880	11.83	663,408	2.73	2,806,337	11.55
2005/06	24,739,254	9,987,508	40.37	8,411,280	34.00	45,620	0.18	2,932,179	11.85	724,340	2.93	2,638,238	10.66
2006/07	27,035,801	10,837,215	40.08	9,060,330	33.51	61,123	0.23	3,044,388	11.26	996,864	3.69	3,035,792	11.23
2007/08	28,637,391	11,593,836	40.49	9,636,670	33.65	93,449	0.33	3,540,815	12.36	730,191	2.55	3,042,341	10.62
2008/09	30,582,700	12,247,319	40.05	10,310,951	33.72	102,358	0.33	3,931,789	12.86	729,445	2.39	3,260,749	10.66
2009/10	31,525,627	12,294,191	39.00	10,569,128	33.53	162,750	0.52	4,048,369	12.84	903,056	2.86	3,548,044	11.25
2010/11	33,224,759	13,158,864	39.61	11,073,996	33.33	178,567	0.54	4,373,819	13.16	806,919	2.43	3,632,505	10.93
2011/12	33,618,042	13,012,645	38.71	11,124,702	33.09	201,243	0.60	4,045,779	12.03	1,046,053	3.11	4,187,532	12.46
2012/13	34,985,042	13,338,215	38.13	11,466,761	32.78	189,526	0.54	4,350,585	12.44	1,037,810	2.97	4,602,058	13.15
2013/14	36,753,212	13,853,621	37.69	11,838,472	32.21	224,221	0.61	4,529,981	12.33	1,271,198	3.46	5,035,633	13.70
2014/15	38,014,388	14,278,578	37.56	12,276,747	32.30	309,030	0.81	4,906,662	12.91	1,402,438	3.69	4,840,846	12.73
2015/16	38,099,208	14,388,408	37.77	12,320,487	32.34	268,026	0.70	4,780,021	12.55	1,543,908	4.05	4,798,271	12.59
2016/17	39,788,985	14,738,027	37.04	12,763,712	32.08	321,866	0.81	4,958,620	12.46	1,874,711	4.71	5,131,962	12.90
2017/18	41,214,192	15,441,474	37.47	13,094,354	31.77	358,787	0.87	4,924,548	11.95	1,907,146	4.63	5,487,796	13.32
2018/19	40,227,790	14,855,789	36.93	12,863,189	31.98	360,936	0.90	5,016,899	12.47	1,833,317	4.56	5,297,573	13.17

#### Crop aftermaths.

Crop aftermath includes all residual materials remaining after harvest, including volunteer plants, regrowth of the main crop, and weeds. Growth, regrowth forage may be grasses, legumes and any other plants that grow after the harvest of cereal or other crop. Crop aftermath is often grazed by livestock, providing additional forage during the post-harvest period and thereby extending the grazing season.

In Ethiopia where livestock are important in the economy and food security, the use of crop aftermath is a common way to use crop passage to enhance livestock feed resources (Fig 4). There is an approximate illustration of crop aftermath as an input to livestock nutrition [37]. This shows that crop aftermath occupies an important role in livestock nutrition across Ethiopia. In many areas, crop aftermath supplies an average of 20% to 30% of total livestock feed resources. Over the past ten years, the percentage contribution of crop aftermath to livestock feed has slightly increased, coinciding with a decline in traditional grazing lands (Fig 2).The findings also illustrate the regional variations: in areas where there is limited grazing land, the contribution of crop aftermath to livestock feed can be greater than or equal to 35% (Fig 1), which is significant in regions where grazing is limited [32]. The trend analysis in Fig 2 and Table 1 also shows a seasonality trend for crop aftermath usage, which is highest during the post-harvest dry season when feed resources are limited [38].

**Fig 5:**
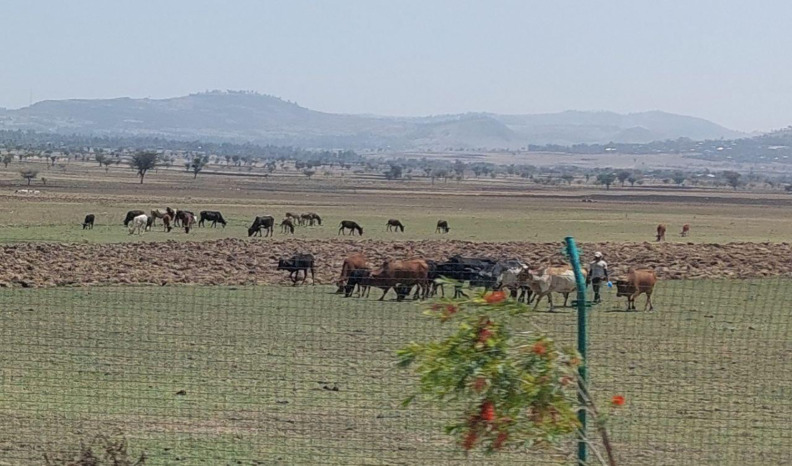
Harvested hay stacks in a pastoral landscape.

Hay usage has steadily increased over the past 15 years, rising from 11.83% in 2004/05 to 12.47% in 2018/19 (Table 1 and Fig 2) reflecting its growing role in maintaining consistent livestock nutrition during feed shortages. Regions like Amhara (20%) and Tigray (23%) rely more on hay, particularly during dry seasons, while surplus production provides additional income for farmers (Table 3). Effective hay storage and management have become crucial strategies for addressing seasonal feed shortages and sustaining livestock productivity in Ethiopia [39]. The steady increase in hay production underscores its importance in livestock feeding strategies. According to Table 1 and Fig 2, hay’s contribution to total livestock feed rose from 11.83% in 2004/05 to 12.47% in 2018/19. This gradual growth reflects a shift toward improved feed storage practices, with more farmers adopting hay harvesting and baling as a strategy to address seasonal feed shortages. In regions like Tigray and Amhara, hay contributes 23% and 20% of livestock feed, respectively (Table 3). This trend underscores hay’s role as a reliable resource during lean periods, particularly in drought-prone areas, while surplus production also provides an additional income source for farmers. The process of harvesting hay and preparing it for baling is shown in Fig 5.

**Table 3:** Animal feeding practices by region (2018/19).

Region	Measurement	Green Fodder	Crop Residue	Improved Feed	Hay	By-Product	Others	Total Holders
Ethiopia		14,855,789	12,863,189	360,956	5,016,899	1,833,317	5,297,573	40,227,723
Tigray	Number	1,207,233	1,172,755	23,344	903,615	205,656	441,667	3,954,270
	Percent	31%	30%	1%	23%	5%	11%	9.80%
Afar	Number	405,093	43,641		5,721	27,757	8,993	491,205
	Percent	82%	9%	0%	1%	6%	2%	1.20%
Amhara	Number	4,297,038	4,191,350	61,128	2,785,782	532,399	1,888,414	13,756,111
	Percent	31%	30%	0%	20%	4%	14%	34.20%
Oromia	Number	5,440,290	4,628,046	79,546	750,670	741,189	1,872,681	13,512,422
	Percent	40%	34%	1%	6%	5%	14%	33.60%
Somale	Number	183,463	113,455	5,631	5,040	17,966	15,604	341,159
	Percent	54%	33%	2%	1%	5%	5%	0.80%
Benshangul_Gumuz	Number	190,371	92,342	2,809	20,233	7,867	14,684	328,306
	Percent	58%	28%	1%	6%	2%	4%	0.80%
SNNP	Number	3,037,052	2,557,312	185,277	542,660	279,168	1,038,890	7,640,359
	Percent	40%	33%	2%	7%	4%	14%	19.00%
Gambella	Number	43,547	5,529	140	213	750	5,383	55,562
	Percent	78%	10%	0%	0%	1%	10%	0.10%
Harari	Number	27,250	36,794	3,061	2,895	10,001	10,832	90,833
	Percent	30%	41%	3%	3%	11%	12%	0.20%
Dire Dawa	Number	24,451	21,964		71	10,563	423	57,472
	Percent	43%	38%	0%	0%	18%	1%	0.10%

In Ethiopia, this process includes loose hay being collected then compressed into bales for storage and transport. Transitioning from loose hay to baled hay enables a more consistent feed supply during dry seasons when pasture is unavailable. Additionally, the sale of baled hay provides an economic boost that supports farming sustainability through improved livestock feeding. From Table 1 and Fig 2, hay has made a contribution to livestock feed of 11.83% in 2004/05 and that percentage has risen to 12.47% in 2018/19, which indicates a rising trend in feeding issues over the dry season when feed is generally limited. Cooperative hay management will not only support livestock during these poor grazing seasons but also improve livelihoods, providing feed to supplement with and also providing additional income sources from a living product [39,40].

#### By-products feeds.

By-product feeds are secondary materials derived from the processing of primary agricultural commodities, utilized as animal feed. These feeds often include materials like bran, hulls, and meals that remain after the extraction of oils, sugars, or other components, providing a cost-effective source of nutrients for livestock [41].

In this study, the by-products feeds showed moderate growth, indicating their increasing adoption as valuable nutritional supplements in livestock diets, which can enhance overall feed quality [42]. By-products contribute approximately 5% to 10% of the overall feed supply, although this varies regionally. These feeds complement traditional sources by supplying essential nutrients vital for livestock during critical periods [37].

The category of improved feed demonstrates a notable upward trend, particularly in the later years, suggesting a shift towards more scientifically formulated nutrition, which is crucial for enhancing livestock productivity and addressing food security challenges [43].

#### Improved feed.

This study shows that improved nutritional feeds are slowly gaining adoption over time. Currently, they account for 0.15% (192,966 t) to 0.90% (630,378 t) of the total feed supply, according to Table 1. Improved forage in Ethiopia includes several key options like the oat-vetch mixture, which yields between 11–18 tonnes per hectare, resulting in approximately 3.7 million tonnes annually. Fodder beet is another vital option, producing 20–25 tonnes per hectare and contributing around 5 million tonnes each year. Desho grass, known for its adaptability, yields 10–15 tonnes per hectare, translating to about 1.5 million tonnes annually. Additionally, tree lucerne provides 8–10 tonnes per hectare, amounting to an estimated 800,000 tonnes per year, while also enhancing soil fertility through nitrogen fixation. These improved forages are essential for boosting livestock productivity and supporting agricultural sustainability in the region [19,44].

Improved feed refers to enhanced formulations of animal feed that are designed to provide better nutritional value, promote growth, and improve overall health and productivity in livestock. These feeds may include additives such as vitamins, minerals, and probiotics to optimize animal performance [45].

While it still makes a small contribution, this increased trend shows a greater uptake of better feed usage, which is very important for getting better productivity on livestock and nutritional improvement in Ethiopia. The increase indicates that livestock keepers are adopting improved feeding practices to enhance livestock productivity and health [46].

In Ethiopia, by-products from agro-industrial sectors, such as flour milling, sugar factories, edible oil processing, abattoirs, and breweries, play a vital role in livestock nutrition. The country has approximately 300 milling houses, producing over 200,000 tonnes of oilseed cakes annually. Breweries contribute about 637,364 tonnes of by-products, which are generally utilized efficiently for animal feed. However, the use of by-products from sugar factories is relatively low, as a significant portion of molasses is redirected for ethanol production, and fibrous by-products are often used as fuel. Additionally, by-products from abattoirs and fisheries are largely wasted, except for those processed at the Addis Ababa Abattoir, which produces meat and bone meal for poultry feed [47].

**Fig 6:**
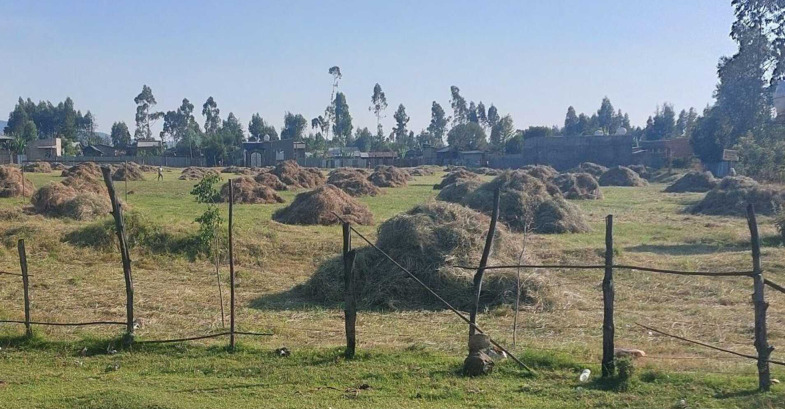
Trends in the percentage of holders using agro-industrial by-products (2004/05 - 2018/19).

Over the years, the number of holders reporting the use of by-products has risen from 2,806,337 in 2004/05 reaching a peak of 5,035,633 in 2013/14, reflecting increasing reliance on these feeds. This peak corresponded to 13.70% of holders utilizing by-products. Despite some fluctuations, the number of holders remained above 4 million, underscoring the critical role of by-products in enhancing livestock nutrition and sustainability in Ethiopia. [48]. The trend in the percentage of holders using agro-industrial by-products (AIBPs) for livestock feeding in Ethiopia is depicted in Fig 6. By the end of the period, the percentage stabilized at about 4.5%, indicating a growing reliance on AIBPs for livestock nutrition. This trend underscores an increasing recognition of the significance of these by-products in enhancing sustainability within the livestock sector [47,48].

### Trends in animal feeding practices among livestock holders

The examination of animal feed sources and the number of livestock holders in Ethiopia from 2004 to 2019 reveals a significant increase in the total number of livestock holders, rising from approximately 24.3 million to about 40.2 million (Table 1). Regarding animal feed usage, green fodder and grazing have remained the predominant sources, with reported usage decreasing from 40.29% in 2004/05 to 36.93% by 2018/19. This decline may indicate diminishing access to natural grazing areas, likely due to land degradation, land put under crop farm and urbanization, which can hinder livestock nutrition [49]. Similarly, the use of crop residues, a critical feed source, slightly decreased from 33.45% to 31.98% during the same period. This decline underscores the challenges livestock holders face in maintaining adequate feed supplies, particularly in rural areas where crop production is closely integrated with livestock farming. [49].

Despite the recognized benefits of improved feeds, their adoption remains alarmingly low, increasing marginally from 0.15% in 2004/05 to only 0.90% in 2018/19. This minimal growth suggests persistent barriers—such as high costs and limited availability—that restrict farmer access to these valuable feed options [49]. In contrast, hay usage has exhibited a positive trend, rising from 11.83% to 12.47%, reflecting increased recognition of hay as a dependable feed resource during dry seasons [4,49]. In contrast, hay usage has exhibited a positive trend, rising from 11.83% to 12.47%, reflecting increased recognition of hay as a dependable feed resource during dry seasons [49].

Overall, the analysis reveals significant shifts in feeding practices and resource availability among livestock holders over the years. These trends highlight the urgent need for targeted interventions aimed at improving access to diverse, high-quality feed sources, measures that are essential for enhancing livestock productivity and strengthening food security in Ethiopia [49].

#### Trends in own holding of animal feed sources.

An analysis of animal feed sources from own holdings in Ethiopia reveals a diverse range of feed types utilized by livestock holders. Many livestock keepers combine communal grazing areas with their private lands, a strategy that maximizes feed availability and allows adaptation to seasonal nutritional variations, ensuring year-round adequate feeding [50,51].

Between 2004/05 and 2018/19, notable shifts occurred in the usage and importance of various feed sources(Table 2). Although green fodder and grazing have historically been significant for livestock nutrition, their proportion declined to 26.3% by 2018/19, likely reflecting challenges such as reduced access to natural grazing lands due to land degradation and urbanization. Crop residues remain the most critical feed resource, accounting for 43.6% of total feed utilized in 2018/19, underscoring the livestock holders’ reliance on agricultural by-products. Despite the proven benefits of improved feeds, their adoption has remained minimal, increasing only to 0.9% by 2018/19, suggesting persistent barriers like cost and limited availability. Hay usage experienced a modest increase, reaching 12.3%, reflecting growing recognition of its value during dry seasons when alternative feeds are scarce. Additionally, the use of by-products rose to 1.9%, indicating an emerging trend towards leveraging agricultural by-products to enhance livestock nutrition.

**Table 2:** Sources of animal feed, feed types and number of holders with their proportion.

Sources	Feed types	2004/05	2008/09	2013/14	2018/19	% proportions as of 2018/19
Own holding	Green Fodder/Grazing	3396590	4446440	5509223	6797213	26.3
	Crops Residue	7410416	9025668	10306195	11251958	43.6
	Improved Feed	27421	63495	181835	239946	0.9
	Hay	1962515	2583005	2877302	3163219	12.3
	By-products	368050	346340	467905	501125	1.9
	Others	1942168	2112516	3370896	3848129	14.9
Purchased	Green Fodder/Grazing	179129	213912	237997	306442	8.9
	Crops Residue	117959	198643	204240	326747	9.5
	Improved Feed	6043	25794	27549	92905	2.7
	Hay	424423	526976	686084	860666	25.0
	By-products	260511	319094	742384	1244612	36.2
	Others	459335	500246	758272	607141	17.7
Communal holdings/government	Green Fodder/Grazing	3530821	3968450	3647533	3434239	93.5
	Crops Residue	99783	145339	101823	108858	3.0
	Improved Feed	1502	3054	2224	2939	0.1
	Hay	110557	112170	148837	90784	2.5
	By-products	5218	5256	4810	2720	0.1
	Others	100899	56922	101123	34428	0.9
From any two or more sources	Green Fodder/Grazing	2668136	3384708	4168188	4028113	61.2
	Crops Residue	491583	813748	1083835	1027702	15.6
	Improved Feed	1421	7949	10462	16732	0.3
	Hay	374107	654119	742957	846767	12.9
	By-products	27776	38272	46304	75016	1.1
	Others	289401	387578	637231	586362	8.9
Other sources	Green Fodder/Grazing	14688	236933	290679	289782	39.5
	Crops Residue	9141	126891	142380	147924	20.2
	Improved Feed	909	920	2151	8415	1.1
	Hay	3277	55278	74801	55464	7.6
	By-products	1853	20231	9795	9844	1.3
	Others	14535	203387	168111	221513	30.2

Overall, analysis of own holdings highlights the crucial role of diverse feed sources in sustaining livestock productivity, while also revealing ongoing challenges that must be addressed to improve access to high-quality feed in Ethiopia.

#### Trends in purchased feed.

Trends in purchased feed utilization among Ethiopian livestock holders show a gradual but significant increase from 2004/05–2018/19. Initially, purchased feed constituted a minor portion of total feed sources, primarily due to limited access and affordability ([50,52]. However, as securing natural grazing and green fodder became more challenging, reliance on purchased feeds grew, reflecting an adaptive shift in feeding strategies and underscoring the increasing importance of commercial feed options despite existing barriers [50,52].

By 2018/19, purchased feed accounted for a substantially larger share of total feed, signalling a strategic shift aimed at ensuring consistent livestock nutrition. This trend reflects a growing recognition of commercial feeds’ role in enhancing productivity, although cost and availability constraints continue to limit widespread adoption. The increasing use of purchased feed demonstrates livestock holders’ adaptive responses to the changing feed availability landscape in Ethiopia [50,52].

### Regional feeding practices

The data presented in Table 3 highlight regional variations in animal feeding practices and feed sources across Ethiopia during the 2018/19 period. Distinct differences emerge between regions, reflecting environmental conditions, agricultural systems, and livestock management strategies.

In the Afar region, an overwhelming 82% of livestock holders primarily rely on green fodder, indicating strong dependence on this feed type, likely due to favourable environmental conditions and abundant natural grazing or pastoral systems [53,54]. Similarly, in Gambella, 78% of holders depend on green fodder, underscoring a comparable pattern of grassland availability [53]. In contrast, Somali region reports 54% reliance on green fodder, which, while substantial, is lower than in Afar and Gambella, suggesting variation in access to suitable grazing lands or differences in livestock management practices [55].

Conversely, urbanized regions such as Harari and Dire Dawa exhibit a markedly different feeding profile. In Harari, 41% of livestock holders depend primarily on crop residues, with Dire Dawa close behind at 38%. This reliance likely reflects limited access to green fodder, driven by urban expansion and agricultural practices favouring food crop production over forage cultivation. Research indicates that increasing urbanization reduces available grazing land, compelling livestock holders to utilize crop by-products more heavily [52]. This trend highlights the challenges faced by farmers in these regions, where urban pressures constrain feed diversity and nutritional quality.

The regions of Amhara, Oromia, and Southern Nations, Nationalities, and Peoples’ (SNNP) present a more balanced feeding strategy. In Amhara, green fodder and crop residues are used by approximately 31% and 30% of holders, respectively, reflecting a dual reliance that optimizes available resources. Oromia demonstrates a higher preference for green fodder at 40%, suggesting comparatively better access to fresh forage. SNNP exhibits similar proportions for green fodder (40%) and crop residues (33%), indicating a diverse feeding system adaptable to seasonal variations and resource availability. Studies on mixed farming systems in Ethiopia have shown that such diversified feeding strategies are critical for coping with feed fluctuations and sustaining livestock productivity.

Overall, these data reveal a clear contradiction between regions favouring green fodder and those reliant on crop residues, emphasizing the importance of region-specific strategies to improve feed availability and livestock nutrition. Addressing these disparities is essential for enhancing livestock productivity and advancing food security in Ethiopia. Tailored interventions that consider local environmental conditions, land use patterns, and agricultural practices will be vital for optimizing animal feeding practices across the country.

#### Correspondence analysis of animal feeding practices in Ethiopia.

Correspondence Analysis (CA) was conducted to explore the distribution and relationships among various feed sources utilized by livestock holders across Ethiopia. This method provides valuable insights into the nutritional strategies prevalent in different regions and highlights the dominant reliance on traditional feed types.

The analysis reveals a strong dependence on traditional feeds such as green fodder and crop residues, which together account for over 65% of feeding practices. Green fodder alone contributes approximately 35% to the chi-squared statistic, while crop residues contribute around 30%. These findings underscore the entrenched role of these feed sources in Ethiopian livestock management, confirming their critical importance in maintaining livestock health and productivity [56].

Significant regional variations in feed utilization emerge from the CA results. Regions like Afar, Gambella, and Somali show predominant use of green fodder, whereas areas such as Harari and Dire Dawa rely more heavily on crop residues. Understanding these regional specifics is crucial for tailoring policies and interventions to local feeding practices Improved feed sources account for only 2% of the chi-squared contribution, reflecting their limited integration into current feeding systems. This low adoption rate highlights substantial barriers—such as cost, availability, and limited awareness that prevent livestock holders from incorporating innovative feeding options into their routines [57]. Addressing these challenges is vital to enhancing livestock productivity and sustainability.

These insights emphasize the need for targeted, region-specific strategies that integrate both traditional and improved feeding approaches. Such integration allows livestock holders to leverage familiar, reliable feed sources while gradually adopting innovations that can improve productivity and food security [58–60].

The Correspondence Analysis (CA) conducted on animal feeding practices in Ethiopia has revealed significant insights into the relationships and distribution of various feed sources utilized by livestock holders. The analysis highlights two critical dimensions that explain a substantial portion of the variance in feeding practices.

The results indicate a strong reliance on traditional feed sources, particularly green fodder and crop residue, which together account for over 65% of livestock feeding practices. Green fodder emerges as the most significant feed source, contributing 35% to the chi-squared statistic, while crop residue follows closely with a 30% contribution. This underscores the entrenched nature of these practices within Ethiopian livestock management, emphasizing the need for region-specific strategies that align with these traditional methods.

Moreover, the analysis highlights a critical gap in the adoption of improved feed, which contributes only 2% to the overall feeding practices. This finding indicates substantial barriers to the acceptance and utilization of innovative feeding practices, suggesting that despite ongoing efforts to introduce better feed options, significant challenges remain that prevent livestock holders from incorporating these alternatives into their routines.

Notable regional variations in feeding practices are also evident. Areas like Afar, Gambella, and Somali show a strong reliance on green fodder, while Harari and Dire Dawa are more dependent on crop residues. This variation emphasizes the necessity for targeted policies that cater to local feeding practices and address the unique challenges faced by livestock holders in different regions.

The CA further quantifies the variation in feeding practices through inertia values: the first dimension explains 50% of the total variation, primarily associated with traditional feeding practices, while the second dimension accounts for 30%, further clarifying the distribution and contrasts among feed types.

The findings from this analysis underscore the urgent need for policy interventions aimed at promoting alternative feed sources, especially improved feeds, to enhance livestock productivity in Ethiopia. Policy recommendations derived from this analysis include promoting education and extension services to increase awareness of improved feeds, improving access to quality feed and veterinary services, and fostering supportive infrastructure that facilitates the adoption of alternative feed sources. These steps are essential for overcoming barriers and achieving sustainable improvements in livestock health and productivity across Ethiopia.

## Conclusions

This study provides significant insights into livestock feeding practices over a 15-year period within the context of Ethiopia’s national feed security, livestock modernization, and food system resilience. The analysis reveals a notable decline in traditional feed sources, specifically green fodder and crop residues, which have historically underpinned livestock nutrition. For instance, green fodder usage decreased from 40.3% to 36.09%, and crop residue use declined from 33.5% to 32.0%, reflecting reductions in reliance on these critical resources. These trends raise concerns about sustaining essential feed resources crucial for the health and productivity of Ethiopia’s rapidly growing livestock population.

Of particular concern is the rapid increase in the number of livestock holders, rising from approximately 24.3 million to 40.2 million, contrasted with a negligible rise in the adoption of improved feeds, from 0.15% to only 0.90%. This disparity highlights persistent structural barriers including limited access, affordability, and awareness, as well as a reliance on traditional feeding practices with often uncertain nutritional value. Without concerted efforts by professional bodies to promote mass adoption of modern feed systems supported by veterinary and experimental research, livestock productivity is unlikely to improve sustainably.

The findings underscore the urgent need for tailored, region-specific policies that address existing gaps in feed production, utilization, and accessibility. Enhancing adoption of improved feeds, investing in feed storage infrastructure, and strengthening capacity building initiatives are critical to achieving sustainable livestock productivity and food security. Moreover, these efforts align with broader national goals of building resilient food systems and fostering climate adaptation within Ethiopia’s livestock sector.

Given that this study relies on historical data, it may not fully capture recent changes and emerging challenges in feeding practices. Future research should examine socio-economic and environmental factors influencing feed adoption, explore the potential of alternative feed resources such as agro-industrial by-products, and assess the impacts of climate variability on feed availability and livestock productivity. Such studies will be essential for developing resilient livestock systems capable of adapting to climate change.

## References

[1] van Huis A. Potential of insects as food and feed in assuring food security. Annu Rev Entomol. 2013;58:563–83. doi: 10.1146/annurev-ento-120811-153704 23020616

[2] Lestari P, Trihadiningrum Y. The impact of improper solid waste management to plastic pollution in Indonesian coast and marine environment. Mar Pollut Bull. 2019;149:110505. doi: 10.1016/j.marpolbul.2019.110505 31442864

[3] Galketi Aratchilage U, Marocco E. Overview of global meat market developments in 2019. Food Agric Organ United Nations; 2020. p. 1–11.

[4] Duguma B, Janssens GPJ. Assessment of livestock feed resources and coping strategies with dry season feed scarcity in mixed crop–livestock farming systems around the gilgel gibe catchment, Southwest Ethiopia. Sustainability. 2021;13(19):10713. doi: 10.3390/su131910713

[5] Bereda A, Yilma Z, Nurfeta A. Dairy production system and constraints in Ezha districts of the Gurage zone, southern Ethiopia. Glob Vet. 2014;12(2):181–6.

[6] Gebreyohanes G, Yilma Z, Moyo S, Okeyo Mwai A. Dairy industry development in Ethiopia: current status, major challenges and potential interventions for improvement. ILRI POSITION Pap Dairy; 2021. p. 1–39.

[7] Mengistu S, Nurfeta A, Tolera A, Bezabih M, Adie A, Wolde-Meskel E. Livestock production challenges and improved forage production efforts in the Damot Gale District of Wolaita Zone, Ethiopia. Adv Agric. 2021;2021.

[8] Food and Agriculture Organization of the United Nations. Livestock production systems spotlight Ethiopia. Rome, Italy: FAO; 2018.

[9] Makkar H, Bediye S, Nemi G. Ethiopian feed industry: current status, challenges and opportunities. Broadening Horizons. 2018;50(1).

[10] Desta AG. Seasonality, balance and copying mechanisms of livestock feed in Northwestern Ethiopia. Cogent Food Agric. 2024;1(10).

[11] O FA. Report on feed inventory and feed balance. Rome, Italy: FAO; 2018.

[12] Ayele J, Tolemariam T, Beyene A, Tadese DA, Tamiru M. Assessment of livestock feed supply and demand concerning livestock productivity in Lalo Kile district of Kellem Wollega Zone, Western Ethiopia. Heliyon. 2021;7(10):e08177. doi: 10.1016/j.heliyon.2021.e08177 34746463 PMC8551507

[13] Worku B, Getnet M, Assaye A. Characterization of cattle production system in East Gojjam zone of Amhara regional state, Ethiopia. Sci Res. 2024;12(1):9–19.

[14] Sewunet Z. Institutions and policies to implement the Ethiopia livestock master plan. Ethiop LMP Br. 2015;5(August 2015):1–4.

[15] Seyoum B, Gemechu N, Harinder M. Ethiopian feed industry: current status, challenges and opportunities. Feed Broadening Horizons. 2018;50(2018):1–9.

[16] Tilahun A, Teklu B, Hoag D. Challenges and contributions of crop production in agro-pastoral systems of Borana Plateau, Ethiopia. Pastoralism. 2017;7(1).

[17] Negash D. Study on compound animal feed demand and animal products, supply, price and marketing in Ethiopia. Biomed J Sci Tech Res. 2022;41(3):32808–17.

[18] ESS. Ethiopian Statistical Service.

[19] FAO and IGAD. Animal Feed Action Plan: sustainably developing livestock-dependent livelihoods in East Africa; 2019. p. 1–50.

[20] Ayele J, Tolemariam T, Beyene A, Tadese DA, Tamiru M. Assessment of livestock feed supply and demand concerning livestock productivity in Lalo Kile District of Kellem Wollega Zone, Western Ethiopia. Heliyon. 2021;7(10). 10.1016/j.heliyon.2021.e08177 PMC8551507 34746463

[21] Amejo AG. Resilience, sustainability, and the role of livestock in rural food systems: a case study from Ethiopia. 2024.

[22] Dar MB. Climate smart livestock feed and forage innovations training workshop. 2022.

[23] FAO. 6 Meat. Agric Outlook 2021-2030; 2021. p. 163–77.

[24] Omotoso OB, Arilekolasi TA, Fajemisin AN. Study on mineral, antinutrient and blood parameters of goats fed molasses treated rice husk. J Food, Nutr Agric. 2019;2(1):10–9.

[25] Tegegne YASMATF. Assessment of sheep production system in Burie district, north western Ethiopia. Glob J Agric Res. 2013;1(2):29–47.

[26] Mekasha A, Gerard B, Tesfaye K, Nigatu L, Duncan AJ. Inter-connection between land use/land cover change and herders’/farmers’ livestock feed resource management strategies: a case study from three Ethiopian eco-environments. Agric Ecosyst Environ. 2014;188:150–62.

[27] Gebremedhin B, Hirpa A, Berhe K. Feed marketing in Ethiopia: results of rapid market appraisal. Improv Product Mark Success Ethiop farmers Proj Work Pap 15 ILRI. Nairobi, Kenya: International Livest Res Institute; 2009;(15). p. 1–64. Available http//www.fao.org/fileadmin/templates/agphome/images/iclsd/documents/wk2_c6_g

[28] Amsalu TAS. Assessment of grazing land and livestock feed balance in Gummara-Rib watershed, Ethiopia. Curr Agric Res. 2014;2(2).

[29] Bachewe F, Minten B, Tadesse F, Taffesse AS. The evolving livestock sector in Ethiopia: growth by heads, not by productivity; 2018. 26 p.

[30] Dijkstrar OO, Groenigen JW van, Spek JW. Diet effects on urine composition of cattle and N2O emissions. 2013. 10.1017/S1751731113000578 23739471

[31] Simeanu D, Radu-Rusu RM. Animal nutrition and productions. Agric. 2023;13(5):1–10.

[32] Derebe B, Worku A, Chanie Y, Wolie A, Rahut DB, The World Bank Group. Harnessing continued growth for accelerated poverty reduction. Heliyon. 2021;7(7):e00797.

[33] Kassahun A, Tadesse T. Psychological dimensions of climate change: perceptions, collective efficacy, and responses in Berehet District, north Shoa, Ethiopia. Clim Change. 2021;176(3).

[34] Hadjipanayiotou M, Labban LM, Kronfoleh AE-R, Verhaeghe L, Naigm T, Al-Wadi M, et al. Studies on the use of dried poultry manure in ruminant diets in Syria. Livest Res Rural Dev. 1993.

[35] Tesfaye A, Chairatanayuth P. Management and feeding systems of crop residues: the experience of East Shoa Zone, Ethiopia. Livest Res Rural Dev. 2007;19(31).

[36] Atuhaire AM, Mugerwa S, Okello S, Lapenga K, Kabi F, Lukwago G. Prioritization of agro-industrial by-products for improved productivity on smallholder dairy farms in the Lake Victoria Crescent, Uganda. Front Sci. 2014;4(1):1–7.

[37] Tibbo M. Sheep production systems and breeding practices for selected zones of Tigray, Northern Ethiopia. Open J Anim Sci. 2019;9(1):1–16.

[38] Ayele S, Abebe G, Tadesse A. The role of traditional grazing management practices in the sustainability of pastoral systems in Ethiopia. Pastor Res Policy Pract. 2021;11(1):1–15.

[39] Mekonnen MM, Hoekstra AY. Global water footprint of animal products: a comprehensive assessment. Ecosystems. 2018;21(3):1–15.31156332

[40] Abebe G. Effective feed management strategies for livestock production: a review. J Anim Sci Technol. 2020;62(1):45–58.

[41] (NRC) NRC. Nutrient requirements of small ruminants: sheep, goats, cervids, and new world camelids. Washington (DC): Natl Acad Press; 2012.

[42] Tadesse M, Abebe G, Mekonnen M. Assessment of feed resources and feeding practices of smallholder dairy farms in the highlands of Ethiopia. Trop Anim Health Prod. 2017;49(5):989–96.28412767

[43] Yami A, Merkel RC. Sheep and goat production handbook for Ethiopia. Addis Ababa: Ethiopia Sheep and Goat Productivity Improvement Program (ESGPIP); 2008.

[44] FAO. Report on feed inventory and feed balance 2018 in Ethiopia; 2018. 138 p.

[45] Bach A, Calsamiglia S, De la Fuente L. Nutritional strategies to improve the health and performance of dairy cows. J Dairy Sci. 2019;102(10):9346–61.

[46] FAO. The state of food security and nutrition in the world 2021: transforming food systems for affordable healthy diets. Rome: FAO; 2021.

[47] FAO, IGAD. Animal Feed Action Plan: sustainably developing livestock-dependent livelihoods in East Africa. 2019.

[48] Bezabih A, Reh S, Moya A. Availability and use of agro-industrial by-products (AIBPs) in livestock feeding in Ethiopia. Front Vet Sci. 2023;123456(10).

[49] Makkar HPS, Siddhuraju P, Becker K. Nutritional and anti-nutritional evaluation of some unconventional feed resources. Anim Feed Sci Technol. 2019;251:85–96.

[50] Mengistu A, Abebe G, Yami A. Assessment of feed resources and feeding practices in smallholder dairy production systems in the highlands of Ethiopia. Trop Anim Health Prod. 2017;49(5):989–96.28412767

[51] Herrero M, Grace D, Njuki J, Johnson N, Enahoro D, Silvestri S, et al. The roles of livestock in developing countries. Animal. 2013;7 Suppl 1:3–18. doi: 10.1017/S1751731112001954 23121696

[52] Duncan AJ, McGowan MM, Dwyer CM. The role of livestock in sustainable food systems. Animal. 2016;10(7):1194–201.

[53] Mengistu A, Kebede G, Assefa G, Feyissa F. Improved forage crops production strategies in Ethiopia: a review. Acad Res J Agric Sci Res. 2016;4(6):285–96.

[54] Mathewos M, Sisay A, Berhanu Y. Grazing intensity effects on rangeland condition and tree diversity in Afar, northeastern Ethiopia. Heliyon. 2023;9(11):e22133. doi: 10.1016/j.heliyon.2023.e22133 38045209 PMC10692821

[55] Tadesse W, Babege K, Wandara S. Assessment on major browse feed resources and determine their chemical composition in Korhaye zone, Somali Region, Ethiopia. Heliyon. 2024;10(22):e40178. doi: 10.1016/j.heliyon.2024.e40178 39605811 PMC11600013

[56] Smith JA, Johnson RL, Williams TH. Innovative approaches to sustainable agriculture. Agric Syst. 2020;178(102740).

[57] Brown TL, Johnson KP. Assessing the role of agroecology in building resilient food systems. Agric Human Values. 2021;38(2):345–57.

[58] Miller SJ. Innovations in crop management: strategies for enhancing yield and sustainability. F Crop Res. 2022;278(108471).

[59] Davis KE, Terblanché SE. Challenges facing the agricultural extension landscape in South Africa, quo vadis? South African J Agric Ext. 2016;44(2):231–47.

[60] Davis RA. Advances in regenerative agriculture: practices and benefits for sustainable farming. Sustainability. 2023;15(4):1234.

